# FLP-1 neuropeptides modulate sensory and motor circuits in the nematode *Caenorhabditis elegans*

**DOI:** 10.1371/journal.pone.0189320

**Published:** 2018-01-02

**Authors:** Ingrid Buntschuh, Daniel A. Raps, Ivor Joseph, Christopher Reid, Alexander Chait, Raubern Totanes, Michelle Sawh, Chris Li

**Affiliations:** 1 Department of Biology, City College of New York, City University of New York, New York, NY, United States of America; 2 Department of Psychology, City College of New York, City University of New York, New York, NY, United States of America; 3 Department of Physics, City College of New York, City University of New York, New York, NY, United States of America; 4 Department of Chemistry & Biochemistry, City College of New York, City University of New York, New York, NY, United States of America; 5 The Graduate Center, City University of New York, New York, NY, United States of America; Brown University, UNITED STATES

## Abstract

Parasitic nematodes infect over one quarter of the population worldwide, causing morbidity in over one billion people. Current anthelmintic drugs are beginning to lose effectiveness due to the presence of resistant strains. We are interested in the role of neuropeptides, which regulate behaviors in all organisms, as another possible target for anthelmintic drugs. FMRFamide-related peptides (FaRPs) are a family of neuropeptides that are conserved throughout the animal kingdom. In particular, nematodes contain the largest family of FaRPs identified thus far and many of these FaRPs are identical among different nematode species; FaRPs in nematodes are collectively referred to as FLPs (FMRFamide-like peptides). However, little is known about the function of these FLPs. We are using the non-parasitic nematode *Caenorhabditis elegans* as a model for examining FLPs in nematodes. *C*. *elegans* contains at least 31 *flp* genes that encode 72 potential FLPs. Among the *flp* genes, *flp-1* is one of the few that is universally found in nematodes. FLP-1 neuropeptides were previously reported to be involved in sensory and motor functions. However, previous alleles of *flp-1* also disrupted a neighboring gene, *daf-10*. To understand the phenotypes of *flp-1*, new alleles that specifically disrupt *flp-1* were characterized. The previously reported locomotory and egg-laying defects were found to be due to loss of *flp-1*, while the osmolarity defect is due to loss of *daf-10*. In addition, loss of *flp-1* and *daf-10* both cause several phenotypes that increase in severity in the double mutants by disrupting different neurons in the neural circuits.

## Introduction

An estimated 1.5 billion people, comprising 24% of the world’s population, are infected with soil-transmitted helminthes [1]. Among the most prevalent infections worldwide, an estimated 807 to 1,221 million people are infected with large roundworms (*Ascaris lumbricoides*) [2], 576 to 740 million people with hookworm [3], and 604 to 795 million with whipworm or *Trichuriasis* [4]. Parasitic nematodes also adversely affect livestock [5] and crops [6]. As parasitic nematodes are difficult to propagate in a laboratory setting, *Caenorhabditis elegans*, a non-parasitic nematode, provides a useful biological model [7].

The World Health Organization’s 2015 list of essential medicines includes eight antifilarials and intestinal anthelmintics [8], which belong to four classes: piperazine, acting as a partial agonist at GABA-gated chloride channels, the benzimidazoles, interacting with β-tubulin to compromise the cytoskeleton, imidazothiazoles/tetrahydropyrimidines, acting as nicotinic receptor agonists, and macrocylic lactones, acting as agonists for the glutamate-gated chloride channel receptors [7,9,10]. All classes of anthelmintics have shown some loss of effectiveness in livestock [11]. In particular, ivermectin, which belongs to the avermectin family of macrocyclic lactones [12] and is one of the three antifilarials on the World Health Organization’s 2015 list of essential medicines [8], is starting to lose its effectiveness against human infections in some areas [13,14], while continuing its loss of effectiveness in livestock [15,16]. To continue to combat the damage caused by parasitic nematodes, there is a need to find other drug targets.

Neuropeptides are involved in most complex behaviors of multi-cellular organisms. Nematodes, specifically, have a larger complement of neuropeptides than other organisms [17,18]. Our focus is on the family of FMRFamide (Phe-Met-Arg-Phe-NH_2_)-related peptides (FaRPs), which was first isolated as a cardioactive agent in mollusks [19]. Since then, a large family of FaRPs, which all share a common RFamide C-terminus, has been identified throughout the animal kingdom where they affect diverse functions [20]. Of particular note, nematodes appear to produce the largest numbers of FaRPs among animals; FaRPs in nematodes are collectively referred to as FLPs (FMRFamide-like peptides). For instance, at least 31 *flp* genes encode FLPs in *C*. *elegans*, where FLPs have been found to have effects on locomotion, egg laying, sleep, and different sensory modalities [18]. As most FLPs have no mammalian counterparts, FLPs and their signaling pathways might provide a useful target for new anthelmintic drugs. With the exception of one receptor in the pond snail [21], FaRPs signal through G protein-coupled receptors [17]. The functions of distinct FaRPs and their signaling pathways are still not completely understood [17,22,23].

*flp-1* is one of the few *flp* genes that is universally found in nematodes [18], making the FLP-1 signaling system an attractive target for anthelmintic drugs. We previously reported that the deletion of *flp-1* caused numerous behavioral defects in *C*. *elegans*, including a reduced response to being touched on the nose, a reduced sensitivity to hyperosmolarity, locomotory defects, and an increased tendency to wander off a food source [22]. *flp-1* mutants were also found to have egg-laying defects [24], which were potentiated in the presence of serotonin [25]. Hence, modulating FLP-1 levels causes effects on mobility and reproduction, thereby increasing its interest as a potential drug target. The *flp-1* gene lies in the first intron of *daf-10*, which was not apparent until the genomic region of *daf-10* was defined [26]. *daf-10* encodes a component of the intraflagellar transport complex A (IFT-A) and is responsible for transport of particles in the cilia of amphidial and phasmid sensory neurons [25–27]. As a result of improper cilia formation, mutations in *daf-10* cause *C*. *elegans* to have reduced sensitivity to hyperosmolarity [28]. Because the initial *flp-1* alleles, *yn2* and *yn4*, also deleted part of *daf-10*, some of the initial behaviors attributed to FLP-1 peptides [22] may be caused by loss of *daf-10* activity. To clarify which behaviors are due to loss of *flp-1*, we characterized the behaviors of recently isolated *flp-1* single mutants and compared these behaviors to those in *daf-10* single and *daf-10 flp-1(yn2)* double mutants. Our results indicate that FLP-1 peptides are involved in nose touch sensitivity, locomotion, and reproduction, making its signaling pathway a potential target for new anthelmintics.

## Materials and methods

### Maintenance of strains

All nematodes were grown and maintained at 20°C according to Brenner [29] with modifications. N2 is the wild-type Bristol strain [29]. Other strains used include: LGIII: *tax-4(p678)*; LGIV: *flp-1(ok2811*, *ok2781*, and *ok2505)*, *daf-10(gk795* and *e1387)*, *daf-10 flp-1(yn2)*.

### PCR and generation of transgenic animals

To determine how the different deletions in the *flp-1* alleles affected the transcripts, we isolated RNA from the different *flp-1* alleles, reverse transcribed the RNA with an oligo dT primer, and amplified the *flp-1* transcript with primers MR28 (5’-GAATTCACTTTATCATGACTCTGC TCTACCAGGTAGGGT-3’) and 33P2 (5’-GGATTCACGATCGAAGTTGTC-3’), which encompasses the region between the initiator and stop codons. Amplified products were excised and the DNA sequence determined commercially (Genewiz).

#### Generation of transgenic lines

To generate constructs to test for rescue of *flp-1* phenotypes, genomic DNA was amplified with primers MR44 (5-CACAGTTCGTTTACATAC-3') and 33P8 (5'-TGCTCCATGGTGGAGAGC-3'), and subcloned into a TA vector (Invitrogen). The resultant clone was digested and subcloned into pB3-SB, which contains a 5.25 kbp fragment of the *flp-1* genomic region, to generate the plasmid pLN/B3-SB, which contains 6 kbp of the *flp-1* promoter, the entire *flp-1* coding region, and 2.3 kbp downstream of the *flp-1* 3’UTR [22]; pLN/B3-SB was digested with HindIII and re-ligated to generate the plasmid pLN/B3H, which contains the same *flp-1* coding and downstream regions as pLN/B3-SB, but only 4 kbp of the *flp-1* promoter region. All transgenic lines, except *ynEx224* and *ynEx228*, were generated as described [30] by co-injecting the pLN/B3-SB plasmid and the SUR-5::GFP marker (injected at 50 ng/μl) [31]; *ynEx224* and *ynEx228* were similarly generated except that the pLN/B3H plasmid was used. Multiple independent transgenic lines were generated for the different *flp-1* and *daf-10* alleles. *Ex* alleles indicate transgenic animals carrying extrachromosomal arrays, while *Is* alleles indicate transgenic animals with integrated transgenes; extrachromosomal arrays were integrated into the genome as described [30] with slight modifications; the insertion site was not mapped. The transgenic lines include: 1) lines injected at plasmid concentrations of 10 ng/μl: *ynEx218*, *ynEx219*, *ynEx220*, *ynEx221*, *ynEx222*, *ynEx223*, 2) lines injected at plasmid concentrations of 20 ng/μl: *ynEx224*, *ynEx225*, *ynEx226*, *ynEx228*, *ynEx229*, *ynIs118;* and 3) lines injected at plasmid concentrations of 50 ng/μl: *ynEx217*, *ynEx227*.

### Behavioral assays

All assays were done on day-old adult hermaphrodites unless otherwise specified. Animals were picked at the fourth larval stage and grown at 20°C for approximately 24 hours prior to performing the assay.

### Locomotion assays

#### Movement on a solid surface

To prepare plates for scoring movement on a solid surface, 100 microliters of *E*. *coli* (OP50/1) was spotted onto a MYOB plate [32] and grown overnight. The next day, a stamp (made by cutting a 1 cm square from the center of a 30 mm Sylgard circle) was applied to the bacterial lawn, leaving an even 1 cm square bacterial lawn. Ten to twenty day-1 adult worms were transferred to the bacterial lawn and left on an Nikon SMZ1000 stereomicroscope stage for 5 minutes to allow the worms to acclimatize to ambient oxygen levels and temperature. The movement of the animals was subsequently recorded at 8X magnification with a Nikon DS-F11 camera using Nikon Instruments Software-Elements Advanced Research (NIS-Elements AR) for 5–10 minutes. The number of body bends was counted for 1 minute starting at the 2-minute interval in the video.

Photographs of day-1 adult worms moving on bacterial food sources were also taken on a Nikon SMZ1000 stereomicroscope with a Nikon DS-F11 camera at 40X magnification. The wavelength and wave height (2x the amplitude) of the tracks in the bacterial lawn were calculated with the NIS-Elements AR imaging program. Five measurements were taken for each animal, averaged, and a mean value for each animal was determined.

#### Movement in liquid

To assess the effects of serotonin on movement [33], individual worms were transferred to a microtiter well containing serotonin solution (5 mg serotonin/ml M9 buffer; Sigma). After approximately 15 minutes in the serotonin solution, the number of body bends completed in 15 seconds was counted as modified from Miller *et al*. [34]. Each individual animal was counted three times and the three values were averaged to give a mean number for each animal.

### Reproductive phenotypes

To determine an egg-laying rate, day-1 adult worms were singly plated and allowed to lay eggs for 2 to 8 hours. The number of eggs laid was counted and the egg-laying rate per hour was calculated. To determine the number of eggs retained in the uterus, day-2 adults were used. 50 microliters of a weak hypochlorite solution (1.2 ml of 6% NaOCl solution (Clorox), 0.5 ml 5 N KOH, and 8.3 ml water) was added to individual rings on the lid of a microtiter plate; individual day-2 worms were transferred to the rings. After the body of the worm was dissolved by the hypochlorite solution, the number of eggs was counted.

### Nose touch

The nose touch response was performed as described [35]. Each day-1 adult was tested three times and the three values were averaged for each animal.

### Osmotic avoidance

The ring assay for osmotic avoidance [28] was done with 10 worms in a 1.5 cm ring of 6 M glycerol (dyed with blue as described [22]). The number of animals within the ring was counted after 10 minutes.

### Wandering

To assay whether animals stay on a food source, 10–20 day-1 adults were placed on a plate with a bacterial food source. The number of animals on the plate was counted at 12 hour intervals for a total of 48 hours.

### Statistical analysis

All statistical analyses were carried out using Prism software (GraphPad). Values from wild-type, mutant, and/or transgenic animals were compared using a one-way ANOVA/Kruskal-Wallis and Tukey or Dunn’s posthoc test, depending on whether the values passed a normality test.

## Results

**Fig 1:**
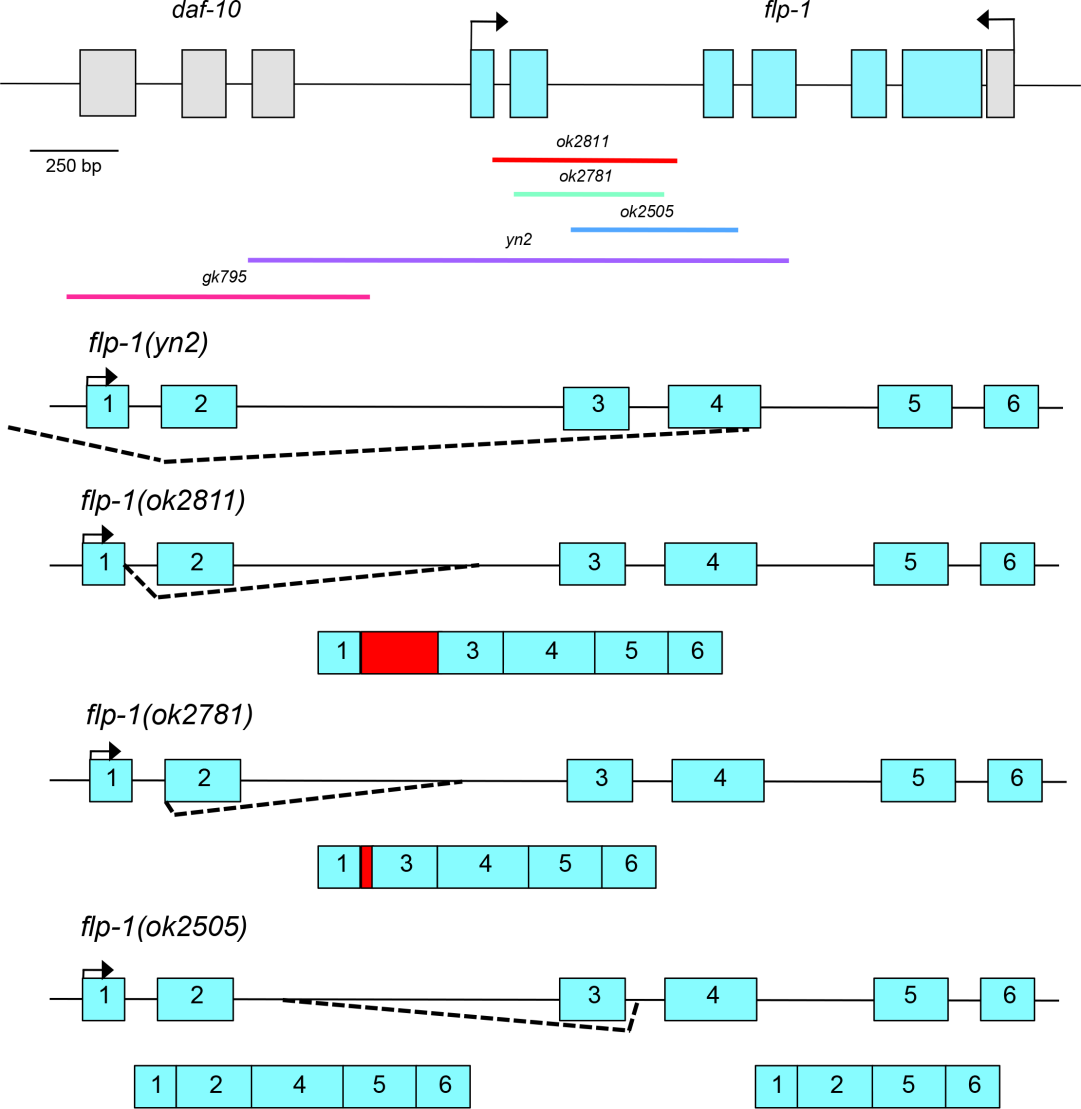
The genomic organization of the *flp-1* gene. *flp-1* consists of six exons encoding seven FLP-1 peptides and one related non-FLP peptide; exons 3 to 6 contain the peptide-coding regions. The *flp-1* allele, *yn2*, is a 1.1 kbp deletion that removes 567 bp of the promoter region and extends through most of exon 4. No *yn2* transcripts were detected by reverse transcription-polymerase chain reaction. The *ok2811* mutation disrupts the splice donor site of exon 1, leading to improper splicing and the inclusion of intronic sequence in the transcript. Only one transcript was detected. The reading frame is disrupted, suggesting that *ok2811* is a null allele. The *ok2781* deletion disrupts the splice acceptor site of exon 2, leading to the use of a cryptic splicing site and inclusion of intronic sequence in the transcript; only one transcript was detected. The reading frame is maintained. The deletion in *ok2505* extends from intron 2 to intron 3; two transcripts are produced, both of which maintain the reading frame but result in the loss of two to four peptides relative to transcript A. *flp-1* and *daf-10* exons are indicated as teal and grey boxes, respectively; only four of the 18 *daf-10* exons are shown. The arrows indicate the direction of transcription; the bars indicate the extent of the deletions.

The *flp-1* gene encodes eight peptides within exons 3 to 6 (Table 1) and is alternatively spliced, giving rise to transcripts A, B, and C [36,37], which encode 7, 6, and 4 distinct FLP-1 peptides, respectively; all transcripts also encode a related, non-FLP-1 peptide [22]. The initial *flp-1* alleles, *yn2* and *yn4*, also deleted part of the *daf-10* gene, making it difficult to determine which phenotypes correspond to loss of *flp-1* and which to loss of *daf-10* (Fig 1). The isolation of additional alleles that only disrupted *flp-1* by the *C*. *elegans* Knockout Consortium allowed us to definitively categorize the phenotypes associated with loss of *flp-1*.

**Table 1:** Peptides encoded by different wild-type *flp-1* transcripts and predictions for FLP-1 peptides produced in mutants.

Peptide*/Transcript/Allele	A	B	C^^^	*ok2811*	*ok2781*	*ok2505*	*yn2*
*KPNFMRFY-NH*_*2*_	x	x	x		x		
AGSDPNFLRF-NH_2_	x				x		
SQPNFLRF-NH_2_	x	x			x	m	
ASGDPNFLRF-NH_2_	x	x			x	m	
SDPNFLRF-NH_2_	x	x	x		x	x	
AAADPNFLRF-NH_2_	x	x	x		x	x	
SADPNFLRF-NH_2_	x	xx	xx		x	x	
(K)PNFLRF-NH_2_	x	x	x		x	x	

*x = no. of copies, italics = non-RFamide peptide, m = additional peptides missing in alternative transcripts

^Although WormBase predicts a transcript C, we have not been able to isolate this transcript by RT-PCR.

The *flp-1 (ok2811*, *ok2781*, *and ok2505)* alleles contain deletions that affect different splice sites or exons within the first three exons (Fig 1), making it difficult to predict *a priori* how transcripts would be affected. Hence, we performed multiple reverse transcription-polymerase chain reactions (RT-PCRs) for each allele, so that all possible transcripts would be isolated, and determined the DNA sequence of the products. For *ok2811* mutants, the deletion affected the splice site at the end of exon 1 and extended past exon 2; the resultant transcript contained intronic sequences and disrupted the reading frame, suggesting that *ok2811* is a null allele; no other transcripts were identified. The *ok2781* deletion affected splicing at the 5’ end of exon 2 and extended into intron 2; the resultant transcript included intronic sequence but maintained the reading frame, suggesting that *ok2781* mutants will have phenotypes similar to wild type; no other transcripts were detected. The *ok2505* deletion extended from intron 2 to intron 3, thereby removing exon 3; however, the resultant transcripts used cryptic splicing sites, resulting in two transcripts in which the coding sequences for two (KPNFMRFY-NH_2_ and AGSDPNFLRF-NH_2_) to four (KPNFMRFY-NH_2_, AGSDPNFLRF-NH_2_, SQPNFLRF-NH_2_, and ASGDPNFLRF-NH_2_) FLP-1 peptides were deleted relative to transcript A. Hence, the three alleles were predicted to represent a presumptive null (*ok2811)*, a presumptive wild type (*ok2781*), and a partial loss-of-function (*ok2505*). Transcription of *daf-10* was not affected by the *flp-1(ok2811*, *ok2781*, *ok2505)* deletions by RT-PCR (S1 Fig).

### Loss of *flp-1* or *daf-10* causes sensory defects

**Fig 2:**
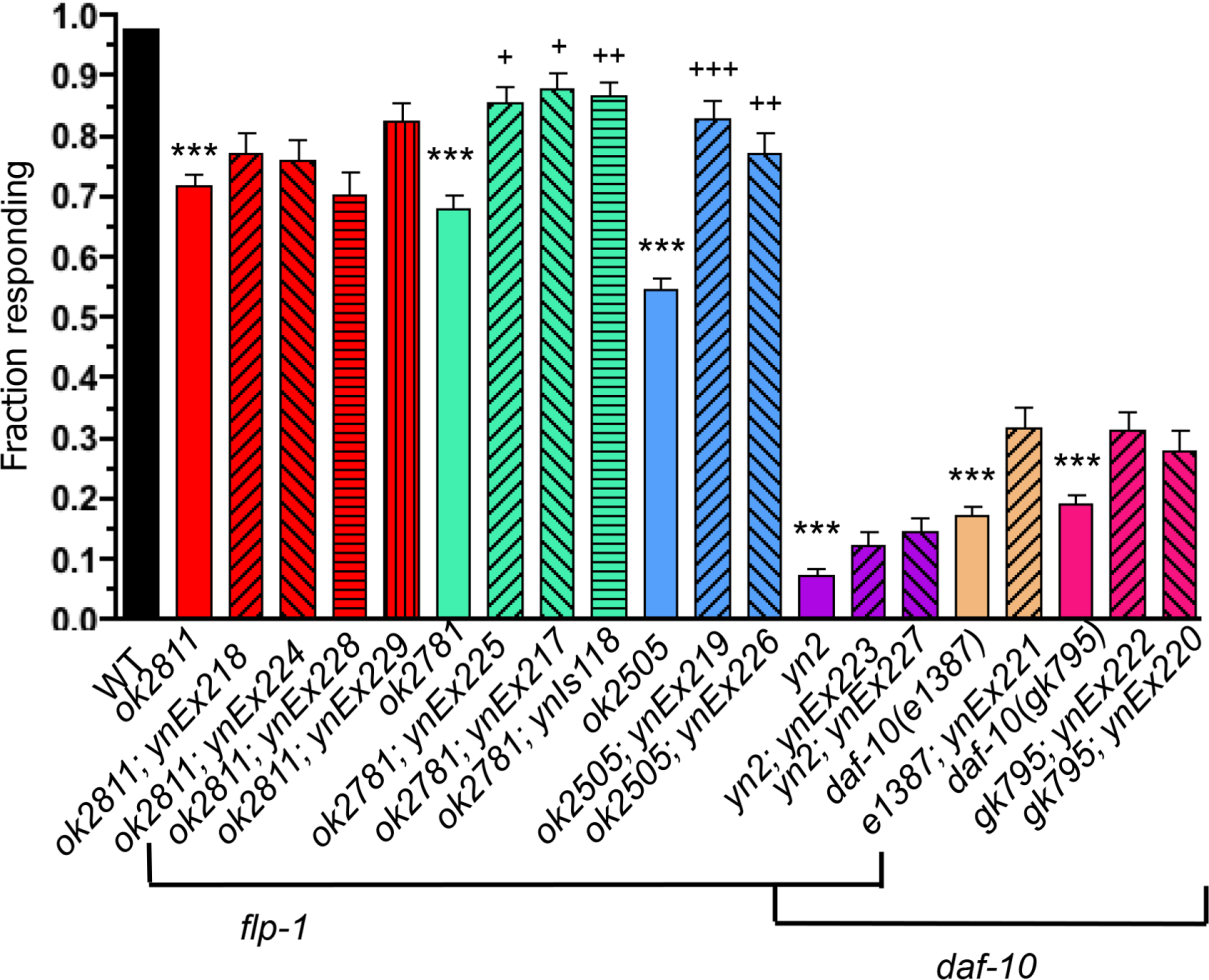
Loss of *flp-1* decreases the nose touch response. All *flp-1* and *daf-10* mutant alleles showed a decreased nose touch response. The decreased nose touch response in *daf-10* mutants was slightly enhanced when *flp-1* was also lost, as shown in the *yn2* double mutants. Note that the *ok2781* allele acts as a partial loss-of-function, despite the peptide coding region being intact. Mean±SEM, at least 72 animals were tested for each strain; each strain was tested in at least six independent trials; solid, colored bars (except black) represent mutant alleles, hatched bars represent transgenic lines. *Ex* alleles indicate a transgenic animal with an extrachromosomal array, while *Is* alleles indicate transgenic animals with integrated transgenes. Significantly different from wild type: ***, p≤0.001, Dunn’s post hoc test; transgenic line significantly different from mutant allele: ^+++^, p≤0.001, ^++^, p≤0.01, ^+^, p≤0.05, Dunn’s posthoc test.

The *daf-10 flp-1(yn2* and *yn4*) double mutants showed defects in two sensory modalities: mechano- and osmosensation. Wild-type animals recoil when touched on their nose (nose touch sensitivity) [35]; this nose touch response is mediated by the ASH multimodal sensory neuron [38]. While wild-type animals respond to nose touch 97% of the time (N = 371), all three *flp-1* mutant alleles showed a significant decrease in the nose touch response (*ok2811*, *ok2781*, and *ok2505* responded 72%, 68% and 55% of the time, respectively), although none of the decreases were as severe as the *yn2* allele, which responded only 7% of the time (Fig 2; Table 2). The nose touch defect was rescued by wild-type *flp-1* in the *ok2781* and *ok2505* mutants. Because *daf-10* mutants have defective sensory cilia, we expected that the mutants would show no nose touch sensitivity, similar to the *yn2* double mutants. Accordingly, the *daf-10* mutants were nose touch insensitive and had slightly, but not significantly higher (17–19%) response rates than *yn2* mutants, and significantly lower response rates than the *flp-1* mutants (Fig 2; Table 2; Dunn’s posthoc test, p<0.0001). These results suggest that loss of *flp-1* slightly exacerbates the sensory loss in the nose touch circuit in *daf-10* mutants.

**Table 2:** Loss of *flp-1* causes multiple defects.

Alleles & Trans-genic Lines	Sensory		Locomotion			Reproduction	
	Nose Touch (Fraction responding)	Osmolarity Response (Fraction within ring)	Body Bends/min	Amplitude x2 (μm)	5HT inhibition	No. of eggs laid/hr	No. of eggs in uterus
wild type	0.97±0.00 (N = 371)	0.97±0.01 (N = 168)	7.543±0.27 (N = 385)	246.6±2.1 (N = 109)	0.63±0.41 (N = 60)	5.3±0.1 (N = 122)	15.91±0.55 (N = 113)
***flp-1***							
*ok2811*	0.72±0.02*** (N = 317)	0.81±0.05 (N = 150)	9.71±0.46*** (N = 196)	307.5±4.3*** (N = 99)	22.61±2.20*** (N = 60)	4.06±0.14*** (N = 113)	17.32±0.61 (N = 105)
*ok2811; ynEx218*	0.77±0.03 (N = 95)		6.30±0.54^+++^ (N = 66)	221.9±4.4^+++^ (N = 26)	0.14±0.13^+++^ (N = 60)	3.26±0.32 (N = 24)	25.47±1.33^+++^ (N = 36)
*ok2811; ynEx224*	0.76±0.03 (N = 85)		7.41±0.48^+^(N = 96)	219.4±5.8^+++^ (N = 20)		2.22±0.37^+++^ (N = 33)	34.65±1.76^+++^ (N = 34)
*ok2811; ynEx228*	0.70±0.04 (N = 97)		6.28±1.02 (N = 18)			3.16±0.22 (N = 35)	11.22±0.88^+++^ (N = 32)
*ok2811; ynEx229*	0.82±0.03 (N = 74)		7.28±0.63 (N = 18)		1.64±0.84^+++^ (N = 60)	3.90±0.29 (N = 36)	24.28±1.62^+++^ (N = 36)
*ok2781*	0.68±0.02*** (N = 321)	0.84±0.04 N = 161)	9.73±0.48** (N = 134)	278.1±3.3*** (N = 100)	32.16±2.14*** (N = 60)	4.38±0.17*** (N = 114)	11.34±0.53*** (N = 111)
*ok2781; ynEx225*	0.85±0.03^+^ (N = 85)		7.56±0.51 (N = 50)	265.8±7.1 (N = 20)			
*ok2781; ynEx217*	0.88±0.03^+^ (N = 75)		7.53±0.76 (N = 40)		3.07±1.21^+++^ (N = 60)	1.48±0.23^+++^ (N = 28)	14.59±1.39 (N = 29)
*ok2781; ynIs118*	0.87±0.02^++^ (N = 117)		5.22±0.67^+++^ (N = 40)	259.2±7.6 (N = 24)	0.63±0.22^+++^ (N = 60)	2.39±0.12^+++^ (N = 37)	11.69±0.88 (N = 35)
*ok2505*	0.55±0.02*** (N = 336)	0.92±0.03 (N = 150)	4.96±0.34*** (N = 161)	257.2±2.6 (N = 105)	16.48±2.26*** (N = 60)	3.65±0.14*** (N = 112)	21.75±0.63*** (N = 104)
*ok2505; ynEx219*	0.83±0.03^+++^ (N = 78)		3.34±0.28 (N = 108)	248.4±7.3 (N = 28)	1.57±0.44^+++^ (N = 60)	1.72±0.28^+++^ (N = 32)	23.88±1.33 (N = 32)
*ok2505; ynEx226*	0.77±0.03^++^ (N = 82)		6.21±0.52 (N = 48)	238.0±7.3 (N = 24)	1.86±0.54^+++^ (N = 60)	3.13±0.22 (N = 36)	17.19±0.86^+^ (N = 36)
***flp-1 daf-10***							
*yn2*	0.07±0.01*** (N = 261)	0.11±0.03*** (N = 140)	2.84±0.52***(N = 129)	292.3±4.2*** (N = 70)	17.77±1.94*** (N = 60)	1.14±0.13*** (N = 73)	19.65±1.04 (N = 43)
*yn2; ynEx223*	0.13±0.02 (N = 72)		0.29±0.10 (N = 45)	221.1±13.5^+++^ (N = 16)	2.33±0.86^+++^ (N = 60)	0.61±0.17^+++^ (N = 30)	26.59±1.91^++^ (N = 27)
*yn2; ynEx227*	0.15±0.02 (N = 84)		1.12±0.45 (N = 42)	225.0±6.1^+++^ (N = 20)	3.03±1.14^+++^ (N = 60)	0.66±0.17 (N = 34)	25.03±1.19^+^ (N = 33)
***daf-10***							
*e1387*	0.17±0.02*** (N = 318)	0.19±0.04***(N = 150)	3.86±0.49*** (N = 73)	224.7±2.7*** (N = 89)	1.76±0.83 (N = 30)	4.37±0.13*** (N = 111)	10.11±0.27*** (N = 111)
*e1387; ynEx221*	0.32±0.04 (N = 85)		1.35±0.30 (N = 26)	212.9±4.5 (N = 22)		4.22±0.20 (N = 30)	13.90±1.27 (N = 30)
*gk795*	0.19±0.02*** (N = 316)	0.17±0.04*** (N = 150)	2.78±0.31*** (N = 85)	242.6±2.6 (N = 91)	1.69±1.21 (N = 30)	4.78±0.10 (N = 112)	11.77±0.32*** (N = 105)
*gk795; ynEx222*	0.31±0.03 (N = 104)		2.35±0.39 (N = 37)	239.7±7.8 (N = 21)		3.85±0.34 (N = 34)	19.66±1.34^+++^ (N = 29)
*gk795; ynEx220*	0.28±0.03 (N = 89)		2.32±0.30 (N = 40)	249.6±8.0 (N = 20)		4.50±0.21 (N = 36)	15.41±0.94 (N = 34)

*Ex* alleles indicate a transgenic animal with a *flp-1* extrachromosomal array, while *Is* alleles indicate transgenic animals with integrated *flp-1* transgenes; different numbers indicate different independent lines; all transgenic lines carry a SUR-5::GFP marker. Significantly different from wild type

***, p≤0.001

**, p≤0.01, Tukey’s post hoc test for all except nose touch and osmolarity, which used a Dunn’s posthoc test; transgenic line significantly different from mutant allele

^+++^, p≤0.001

^++^, p≤0.01

^+^, p≤0.05, Tukey’s post hoc test for all except nose touch and osmolarity, which used a Dunn’s posthoc test and body bends, which used a Kruskal-Wallis test.

Wild-type animals are highly sensitive to environmental conditions. For instance, *C*. *elegans* avoids areas of high osmolarity [28] and will avoid crossing a high osmolarity ring (97% within ring; N = 168; no. trials = 16; Table 2). *yn2* double mutants are insensitive to high osmolarity (11% within ring; N = 140; no. trials = 14; [22]). Similarly, *daf-10* single mutants showed no response to high osmolarity (19% and 17% within ring for *e1387* and *gk795*, respectively; N = 150; no. of trials = 15; Table 2). By contrast, all *flp-1* single mutants were sensitive to high osmolarity (81%, 84%, and 92% within ring for *ok2811* (N = 150; no. of trials = 15), *ok2781* (N = 161; no. of trials = 16), and *ok2505* (N = 150; no. of trials = 15), respectively), indicating that the osmolarity defect in *yn2* double mutants is due to loss of *daf-10* alone.

### Increased levels of FLP-1 peptides decrease locomotion

**Fig 3:**
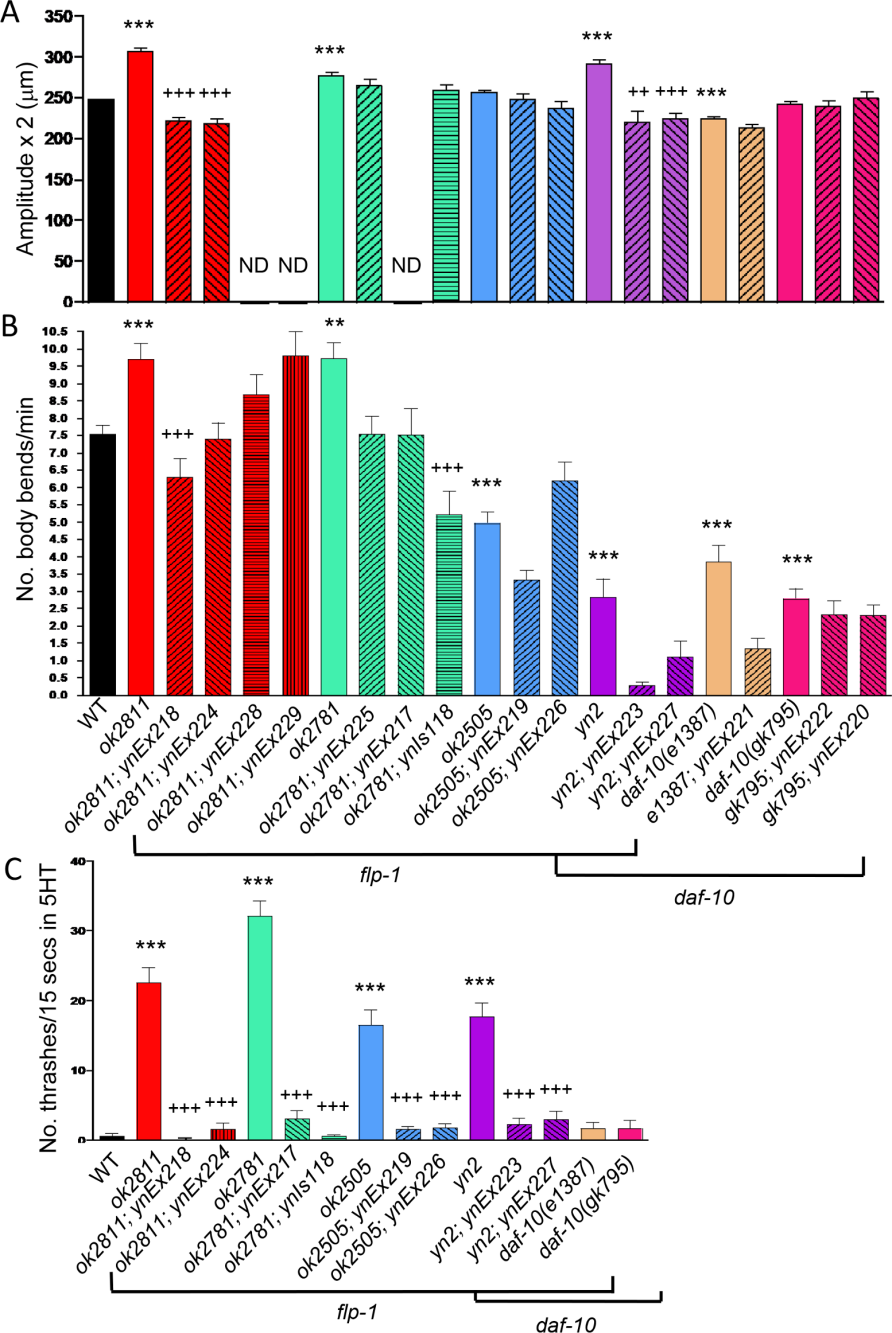
FLP-1 peptides modulate locomotion. Loss of *flp-1* causes hyperactivity, while overexpression of *flp-1* decreases activity. Both the amplitude of the sinusoidal waveform (A) and movement rate (B) were affected. In addition, while exogenously applied serotonin inhibits swimming in wild-type animals, *flp-1* mutants continued to swim in the presence of serotonin (C). *daf-10* IFT-A mutants showed a decreased locomotory activity and were responsive to the inhibitory effects of serotonin. Mean±SEM, solid, colored bars (except black) represent mutant alleles, hatched bars represent transgenic lines. *Ex* alleles indicate a transgenic animal with an extrachromosomal array, while *Is* alleles indicate transgenic animals with integrated transgenes. Significantly different from wild type: ***, p≤0.001, **, p≤0.01, Tukey’s post hoc test; transgenic line significantly different from mutant allele: ^+++^, p≤0.001, ^++^, p≤0.01, Tukey’s post hoc test, each strain was tested in at least three independent trials.

In its natural habitat, *C*. *elegans* moves between soil and liquid, depending on the moisture in the soil. To mimic these conditions in the laboratory, we assayed crawling and swimming movement of animals on an agar surface, which is analogous to soil, and in liquid, respectively. We previously reported that *yn2* double mutants showed hyperactivity and a larger waveform when crawling compared to wild type, while overexpression of *flp-1* inhibited movement and showed a smaller waveform [22]. Here, we further dissected the locomotory patterns of *yn2* double and *flp-1* single mutants. Movement of *yn2* double mutants is bimodal. When *yn2* double mutants are measured only in their locomotory phase, they demonstrate hyperactivity as reported in Nelson et al. [22] and showed a larger waveform than wild type (Fig 3A; Table 2). However, *yn2* double mutants also showed long periods of inactivity interspersed with rapid movements; for instance, 59% of *yn2* double mutants (n = 134) showed no forward movement during our 1 minute analysis, whereas only 7% of wild-type animals (n = 358) showed no forward movement. Therefore, if movement was measured as activity for a specific length of time, such as one minute, *yn2* double mutants showed hypoactivity, similar to *daf-10* mutants (Fig 3B; Table 2). The single *flp-1* mutants, *ok2811* and *ok2781*, showed increased crawling mobility and larger waveforms (Fig 3A and 3B; Table 2). Hence, the locomotion phenotype of *yn2* double mutants is the combined result of the lack of sensory input due to the *daf-10* mutation and the hyperactivity due to loss of FLP-1 peptides. In contrast to the hyperactive movements of *ok2811* and *ok2781* mutants, *ok2505* mutants were very sluggish (Fig 3B; Table 2) and showed decreased movement. Because *ok2505* mutants are predicted to express only a subset of the FLP-1 peptides, these results suggest that different FLP-1 peptides have different effects on the locomotory circuit.

Exogenous serotonin inhibits locomotion in *C*. *elegans* [33]. Wild-type animals exposed to exogenous serotonin stop swimming within minutes (Fig 3C; Table 2). *yn2* double mutants were insensitive to the serotonin-induced sluggishness [22]. To determine whether this serotonin-insensitivity is due to loss of *flp-1* or *daf-10*, single *flp-1* and *daf-10* mutants were similarly exposed to exogenous serotonin. Like wild-type animals, *daf-10* mutants stopped swimming in the presence of serotonin. By contrast, all *flp-1* mutants continued swimming in the presence of serotonin (Fig 3C). The swimming phenotype was rescued by a wild-type *flp-1* fragment. Hence, the serotonin-insensitivity is due to loss of FLP-1 peptides.

**Fig 4:**
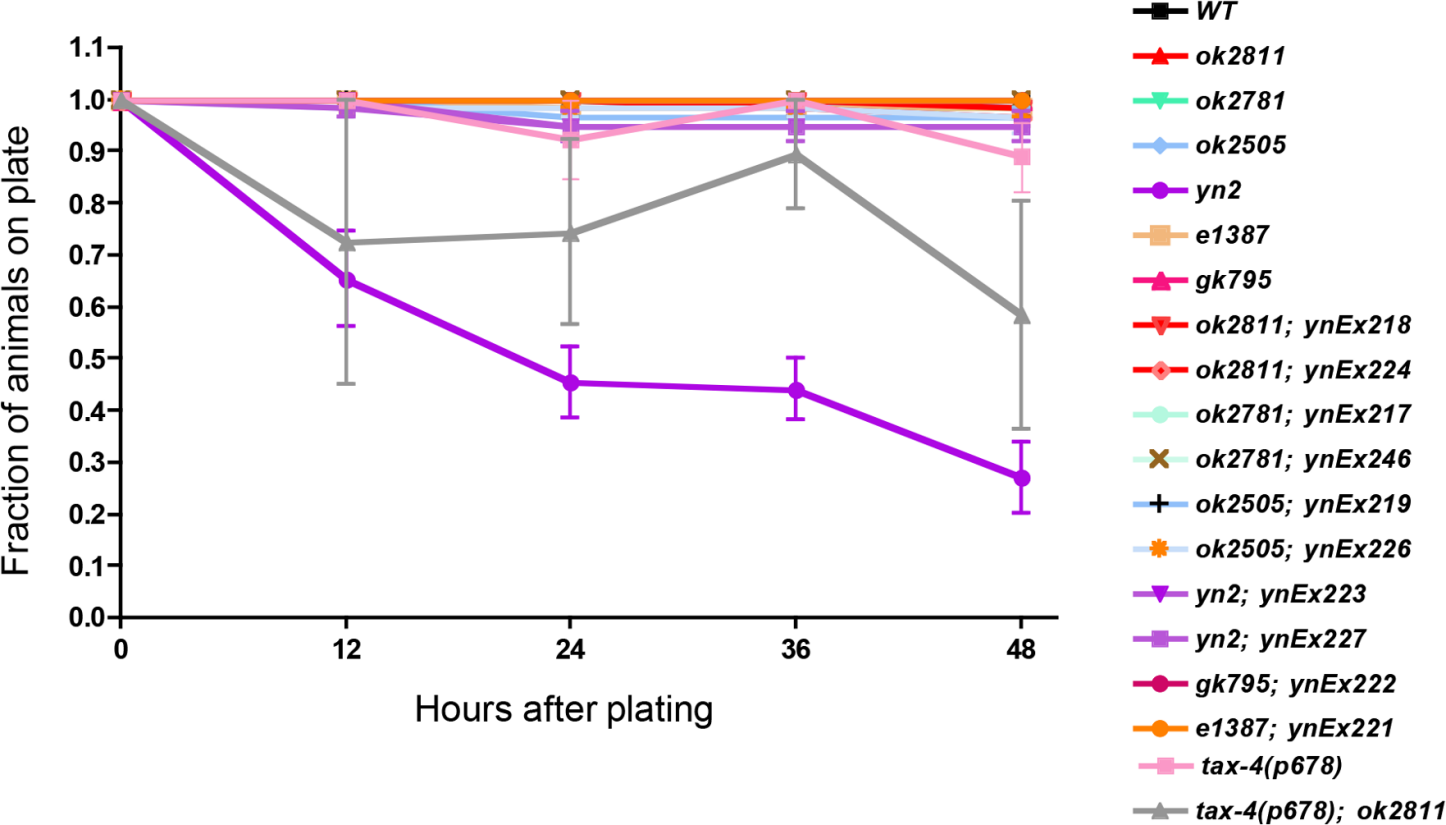
Loss of sensory input and *flp-1* signaling is responsible for the wandering behavior. Wild-type animals remain on a bacterial food source; animals that move off a food source and do not return are considered wanderers. *daf-10 flp-1* and *tax-4; flp-1* double mutants are wanderers, while the single *flp-1*, *daf-10*, and *tax-4* mutants do not wander. For the *tax-4(p678)* and *tax-4(p678); ok2811* strains, animals were not scored at each time point, giving rise to some fluctuation in the data. 3–5 trials for each strain; at least 30 animals were tested for each strain.

*yn2* double mutants, have an additional sensory-motor phenotype, which we termed wandering. Wild-type animals stay on a bacterial food source; however, *yn2* double mutants wander off the food source and up the side of the plate, where they die [22]. The wandering phenotype is not due to the loss of chemosensory input, as *yn2* double mutants can chemotax to a bacterial food source [22]; furthermore, *daf-10* single mutants, which have impaired sensory responses, and *flp-1* mutants did not exhibit wandering (Fig 4). To determine whether the wandering phenotype was a result of loss of sensory input and *flp-1* signaling, we mated *flp-1(ok2811)* mutants with a second mutant that has decreased sensory responses, *tax-4*. *tax-4* encodes a subunit of a cyclic nucleotide-gated channel that is homologous to the vertebrate rod photoreceptor cGMP-gated channel and that is found in a subset of sensory neurons; loss of *tax-4* results in defects in several sensory behaviors [39]. The *tax-4; flp-1* double mutants showed a wandering behavior, although not as robustly as the *yn2* mutants. We suggest that wandering is the result of a synergistic interaction between loss of sensory input and FLP-1 signaling in downstream circuits.

### Increased levels of FLP-1 peptides decrease egg-laying rates

**Fig 5:**
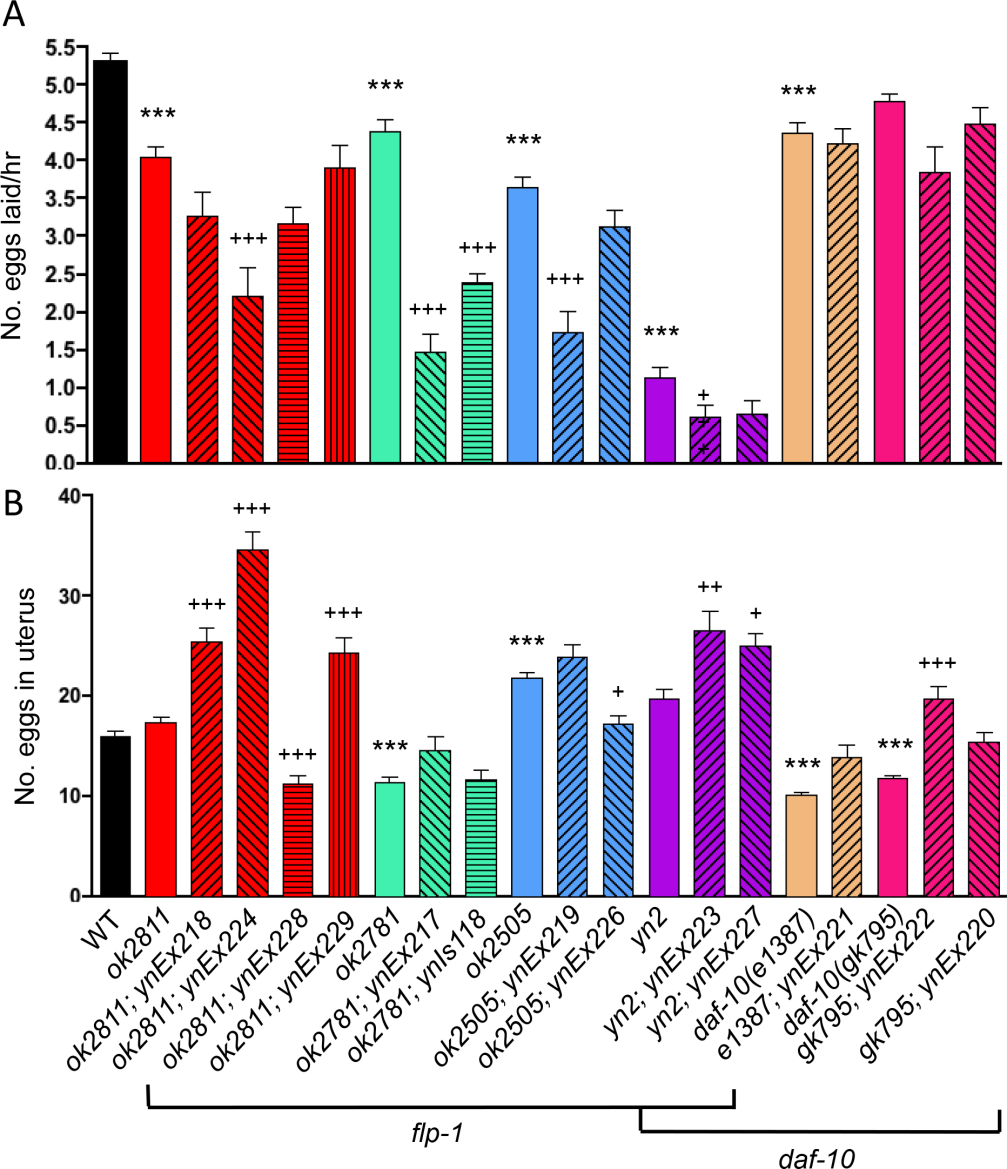
Modulation of *flp-1* affects reproductive functions. Loss and overexpression of *flp-1* decreased egg-laying rates (A), suggesting that different FLP-1 peptides modulate the egg-laying circuit differently. Loss and overexpression of *flp-1* generally increased the number of eggs retained in the uterus (B), again suggesting that individual FLP-1 peptides affect egg retention differently. Loss of *daf-10* IFT-A function also affects reproduction. Mean±SEM, at least 43 or 24 mutants or transgenic mutants, respectively, were tested in at least 3 independent trials; solid, colored bars (except black) represent mutant alleles, hatched bars represent transgenic lines. *Ex* alleles indicate a transgenic animal with an extrachromosomal array, while *Is* alleles indicate transgenic animals with integrated transgenes. Significantly different from wild type: ***, p≤0.001, Tukey’s post hoc test; transgenic line significantly different from mutant allele: ^+++^, p≤0.001, ^++^, p≤0.01, ^+^, p≤0.05, Tukey’s post hoc test. ND = not determined.

Wild-type animals lay between 5–8 eggs per hour [22], but the rate of egg laying is highly affected by environmental stresses and the animal’s metabolic rate. For example, under harsh environmental conditions, such as low food availability, high osmolarity, and high vibration frequency, egg-laying rates are decreased [40]. *yn2* double mutants had a severely decreased egg laying rate of roughly one egg per hour (Fig 5A; Table 2). All of the single *flp-1* mutants also showed a significantly decreased egg-laying rate, although none showed phenotypes as severe as that in the *yn2* double mutants. Rather than being rescued by microinjection of a wild-type *flp-1* fragment, the transgenic *flp-1* mutants as well as the *yn2* double mutants showed an even more severe egg-laying defect. The *daf-10(e1387)* allele also showed a significantly decreased egg laying rate, which was not seen in the *daf-10(gk793)* allele.

The decreased egg-laying rate in *flp-1* mutants can result from several variables: mutants are generating fewer eggs, a sensor controlling the number of eggs in the uterus is defective, or muscles are not responsive to the HSN and VC motor neuron output [40]. To determine whether the decreased egg-laying rate was due to a decreased number of available eggs for release, we counted the number of eggs in the uterus. Wild-type animals have 10–15 eggs in their uterus at any one time [40,41], although we had slightly higher numbers in our current assays (2 day adults: 15.91±0.55 (N = 113); Fig 5B; Table 2). The *yn2* double and *ok2811* null mutants did not show any significant differences compared to wild type. By contrast, the *ok2781* and *ok2505* mutants showed significantly fewer and significantly greater numbers of retained eggs, respectively (Fig 5B; Table 2). Microinjection of the *flp-1* fragment significantly increased egg retention in all *flp-1* mutants (Fig 5B; Table 2). Loss of *daf-10* significantly decreased egg retention but only slightly decreased egg-laying rates. Overexpression of *flp-1* in the *daf-10* mutants, however, increased egg retention.

## Discussion

In identifying new drug targets for anthelmintic drugs, peptide signaling systems that inhibit locomotion or reproduction are attractive targets. FLP-1 neuropeptides were previously found to have roles in locomotion and egg laying [22,24]. Because the *flp-1* gene lies within the first intron of the *daf-10* gene and the initial *flp-1* alleles, *yn2* and *yn4*, affected the activity of both *flp-1* and *daf-10*, we examined more recently isolated single *flp-1* alleles for sensory, locomotory, and reproductive defects. Surprisingly, although the *ok2781* mutants were predicted to have wild-type behavior because no peptide coding regions are lost, the mutants acted more as loss-of-function mutants, more often showing phenotypes similar to or less severe than *ok2811* mutants, which are the presumed *flp-1* null mutants. These results suggest that some FLP-1 peptides are still expressed, albeit at low levels, in *ok2781* mutants. The phenotypes of *ok2505* mutants varied greatly compared to *ok2811* mutants, sometimes showing more severe phenotypes (e.g., nose touch response, crawling rate, resistance to serotonin, and number of eggs retained in the uterus) and sometimes acting similar to wild-type (e.g., waveform), suggesting that the different FLP-1 peptides affect neural circuits differently. Because *ok2505* mutants expressed only a subset of FLP-1 peptides, we cannot distinguish at this time which FLPs promote and which inhibit different behaviors.

DAF-10 IFT-A is required for proper cilia formation in amphid and phasmid neurons in *C*. *elegans* [26,42]. The cilia, which are located at the distal endings of the dendrites, contain the molecular machinery for sensory transduction and translating information from the environment [26]. The loss of DAF-10 IFT-A causes the animals to have defects in osmo- and chemosensitivity [42], whereas *flp-1* single mutants have no osmolarity defect. Hence, the osmolarity defect previously reported in the *yn2* double mutants as caused by *flp-1* [22] was actually due to the loss of *daf-10*.

The ASH sensory neuron, whose sensory cilia are functionally dependent on DAF-10 activity, mediates two distinct sensory stimuli: nose touch and avoidance of noxious chemicals and hyperosmolarity; activation of ASH results in signaling to AVA interneurons, the driver cells for backward locomotion, causing the animal to initiate a reversal [43]. However, nose touch and avoidance to noxious chemicals activate distinct cellular signaling pathways, so that loss of one ASH sensory modality does not imply loss of other ASH sensory modalities [43]. *yn2* double mutants were not responsive to nose touch or hyperosmolarity [22], whereas *flp-1* mutants were nose touch insensitive, but hyperosmolarity sensitive. *flp-1* is expressed in the AVA interneuron [22]. Because the *yn2* double mutant has a more severe nose touch insensitivity than the single mutants alone, we propose that the enhanced nose touch defect in the *yn2* double mutants is due to the reduction of the ASH signal due to the faulty cilia in *daf-10* mutants, compounded by the reduction of downstream AVA interneuron signaling caused by the loss of *flp-1*. Alternative signaling pathways mediate the hyperosmolarity response in *flp-1* mutants.

Disrupted sensory input and exogenous serotonin decrease locomotion [33,44]. The locomotory activity of *yn2* double mutants was further characterized. The *yn2* double mutants displayed extended periods of inactivity, which we attribute to loss of *daf-10*, as the *daf-10* single mutants show a similar phenotype. When the double mutants were active, the speed with which they moved [22] and the sinusoidal waveform they exhibited were much greater than that of wild type. *flp-1* single mutants, *ok2811* and *ok2781*, were also hyperactive and had exaggerated waveforms (Fig 3A and 3B; Table 2). Hence, the speed and waveform defects are due to loss of *flp-1*. The slowed speed and wild-type waveform of *ok2505* mutants suggests that different FLP-1 peptides have different effects on locomotory circuits. Our results do not differentiate between whether the lost FLP-1 peptides in *ok2505* mutants activate locomotion or the remaining peptides inhibit locomotion. Both the *yn2* double mutants and *flp-1* single mutants were insensitive to the serotonin-induced decreased locomotion, whereas the *daf-10* single mutants stop swimming in the presence of exogenous serotonin. Collectively, our results indicate that the overall activity of FLP-1 peptides, like serotonin, is to inhibit locomotory circuits. These *flp-1* locomotory circuits can over-ride the locomotory circuits in which serotonin is active, as *flp-1* mutants continue to swim in the presence of serotonin while wild-type and *daf-10* single mutants stop swimming.

When *C*. *elegans* are starved, they become more active as they constantly forage for food [45]. Often times, starved animals wander off the agar medium and climb onto the side of plates where they die, a phenotype we refer to as wandering. *flp-1* and *daf-10* single mutants do not wander. By contrast, the *yn2* double mutants, which can sense food [22], climb onto the side of plates even in the presence of food. We suggest that wandering in *yn2* double mutants is a synergistic effect caused by the loss of sensory input due to the knockout of *daf-10* and the loss of downstream interneuron signaling by *flp-1*. Similarly, decreasing sensory input by a different mutation, such as *tax-4*, in *flp-1* mutants also caused a wandering phenotype. Because *tax-4* is only expressed in a subset of amphidial neurons [39] and DAF-10 is expressed in all amphidial neurons, we predicted and found that the wandering phenotype was not as severe in *tax-4; flp-1* double mutants.

The *yn2* double mutant has a severe egg-laying defect [22]. Because only one *daf-10* allele showed a decreased egg laying rate, we conclude that loss of amphidial and phasmid sensory input affects egg-laying rates only slightly. By contrast, *flp-1* single mutants exhibited a significantly decreased egg-laying rate, albeit not as severe as the double mutant. The egg-laying neural circuit consists of two motor neurons, HSN and VC, which synapse onto the vulval muscles to cause muscle contraction and egg release [46]. Both neurons receive little synaptic input from other cells: VC neurons receive input from HSN and other VC neurons, while HSN neurons receive input from the VCs, two interneurons, and PLM, a body touch mechanosensory neuron [46]. Because *flp-1* is not expressed in the egg-laying neural circuit, these results suggest that FLP-1 peptides act extra-synaptically to modulate the egg-laying neural circuit. Furthermore, because both loss and overexpression of FLP-1 peptides decrease egg-laying rates, either different FLP-1 peptides have different effects on the egg-laying circuit or high levels of FLP-1 peptides, as in the transgenic animals, cause activation of additional circuits to decrease egg laying.

## Conclusions

Sensory neurons send signals to interneurons, which in turn signal to motor neurons, resulting in an animal responding to a change in the environment [47]. DAF-10 IFT-A activity in amphidial and phasmid sensory neurons is necessary for ciliogenesis and, therefore, sensory function. *flp-1* is expressed in many interneurons [22], which serve to transmit information from the sensory neurons to the motor neurons. Hence, decreased DAF-10 IFT-A activity affects behaviors that are dependent on sensory information, such as osmolarity and nose touch responses, locomotion, and egg laying; with the exception of osmolarity, these sensory deficits are exacerbated when *flp-1* signaling is lost in the interneurons. As FLP-1 peptides are not expressed in the egg-laying neural circuit, the FLP-1 peptides must be acting extra-synaptically to influence egg-laying rate. Furthermore, different FLP-1 peptides must be affecting egg-laying circuits differentially as both increased and decreased FLP-1 peptide levels result in decreased egg-laying rate. Determining the function of specific FLP-1 peptides awaits future studies.

The wandering behavior is the result of a synergistic effect of loss of both sensory function and downstream signaling through FLP-1 peptides. Wandering animals are able to chemotax to food, but wander off food within a few hours. Chemoattraction to food is dependent on amphidial neurons; however, loss of amphidial function in *daf-10* mutants does not result in wandering animals, suggesting that a different pathway that utilizes FLP-1 peptides to sense food is operating. Loss of both pathways results in wandering.

## Supporting information

S1 File Supporting material & methods. RNA was isolated from populations of wild type (N2), *daf-10 flp-1(yn2)*, and *flp-1(ok2811*, *ok2781*, and *ok2505)* mutants with Trizol (Ambion) and reverse-transcribed with an oligo-dT primer according to the manufacturer’s protocol (Promega). Synthesized cDNA was amplified with MR23 (5’-CTGCAGATAACAAAGTTTACTTG) and D10-2 (5’-GACGTACTGTCACTGACCAAATCC) or with MR38 (5’-CGCACATTTGCATCAAGAG) and D10-2. Products were electrophoresed on an 0.8% agarose gel and visualized with a UV transilluminator (UVP gel-doc system).
(DOCX)

S1 Fig Expression of *daf-10* transcripts is not affected in *flp-1(ok2811*, *ok2781*, *ok2505)* mutants. RNA from wild type (WT), *flp-1* single, and *daf-10 flp-1(yn2)* double mutants was isolated and reverse transcribed with an oligo-dT primer. The cDNA was amplified with two primer pairs, one set (MR23/D10-2) in which one primer is contained within the *yn2* deletion, but not within the *flp-1* single deletion mutations (756 bp, arrow), and one set (MR38/D10-2) in which the primers are not contained within the *yn2* deletion or any *flp-1* single deletions (283 bp, chevron). The larger products (722 bp) in the MR38/D10-2 amplification (red asterisks) were due to DNA contamination. M = molecular weight markers.
(TIF)
