# Supplementary material for: FLP-1 neuropeptides modulate sensory and motor circuits in the nematode Caenorhabditis elegans
Source: PLoS One. 2018 Jan 2;13(1):e0189320. doi: 10.1371/journal.pone.0189320 (PMC5749679; doi:10.1371/journal.pone.0189320)
Supplement: S1 File — RNA was isolated from populations of wild type (N2), daf-10 flp-1(yn2), and flp-1(ok2811, ok2781, and ok2505) mutants with Trizol (Ambion) and reverse-transcribed with an oligo-dT primer according to the manufacturer’s protocol (Promega). Synthesized cDNA was amplified with MR23 (5’-CTGCAGATAACAAAGTTTACTTG) and D10-2 (5’-GACGTACTGTCACTGACCAAATCC) or with MR38 (5’-CGCACATTTGCATCAAGAG) and D10-2. Products were electrophoresed on an 0.8% agarose gel and visualized with a UV transilluminator (UVP gel-doc system). (DOCX) [file pone.0189320.s001.docx]

Supporting Information

Supporting Material & Methods

RNA was isolated from populations of wild type (N2), *daf-10 flp-1(yn2)*, and *flp-1(ok2811, ok2781,* and *ok2505)* mutants with Trizol (Ambion) and reverse-transcribed with an oligo-dT primer according to the manufacturer’s protocol (Promega). Synthesized cDNA was amplified with MR23 (5’-CTGCAGATAACAAAGTTTACTTG) and D10-2 (5’-GACGTACTGTCACTGACCAAATCC) or with MR38 (5’-CGCACATTTGCATCAAGAG) and D10-2. Products were electrophoresed on an 0.8% agarose gel and visualized with a UV transilluminator (UVP gel-doc system).

Figure S1. Expression of *daf-10* transcripts is not affected in *flp-1(ok2811, ok2781, ok2505)* mutants. RNA from wild type (WT), *flp-1* single, and *daf-10 flp-1(yn2)* double mutants was isolated and reverse transcribed with an oligo-dT primer. The cDNA was amplified with two primer pairs, one set (MR23/D10-2) in which one primer is contained within the *yn2* deletion, but not within the *flp-1* single deletion mutations (756 bp, arrow), and one set (MR38/D10-2) in which the primers are not contained within the *yn2* deletion or any *flp-1* single deletions (283 bp, chevron). The larger products (722 bp) in the MR38/D10-2 amplification (red asterisks) were due to DNA contamination. M=molecular weight markers.
