# Supplementary figures and images for: FLP-1 neuropeptides modulate sensory and motor circuits in the nematode Caenorhabditis elegans
Source: PLoS One. 2018 Jan 2;13(1):e0189320. doi: 10.1371/journal.pone.0189320 (PMC5749679; doi:10.1371/journal.pone.0189320)

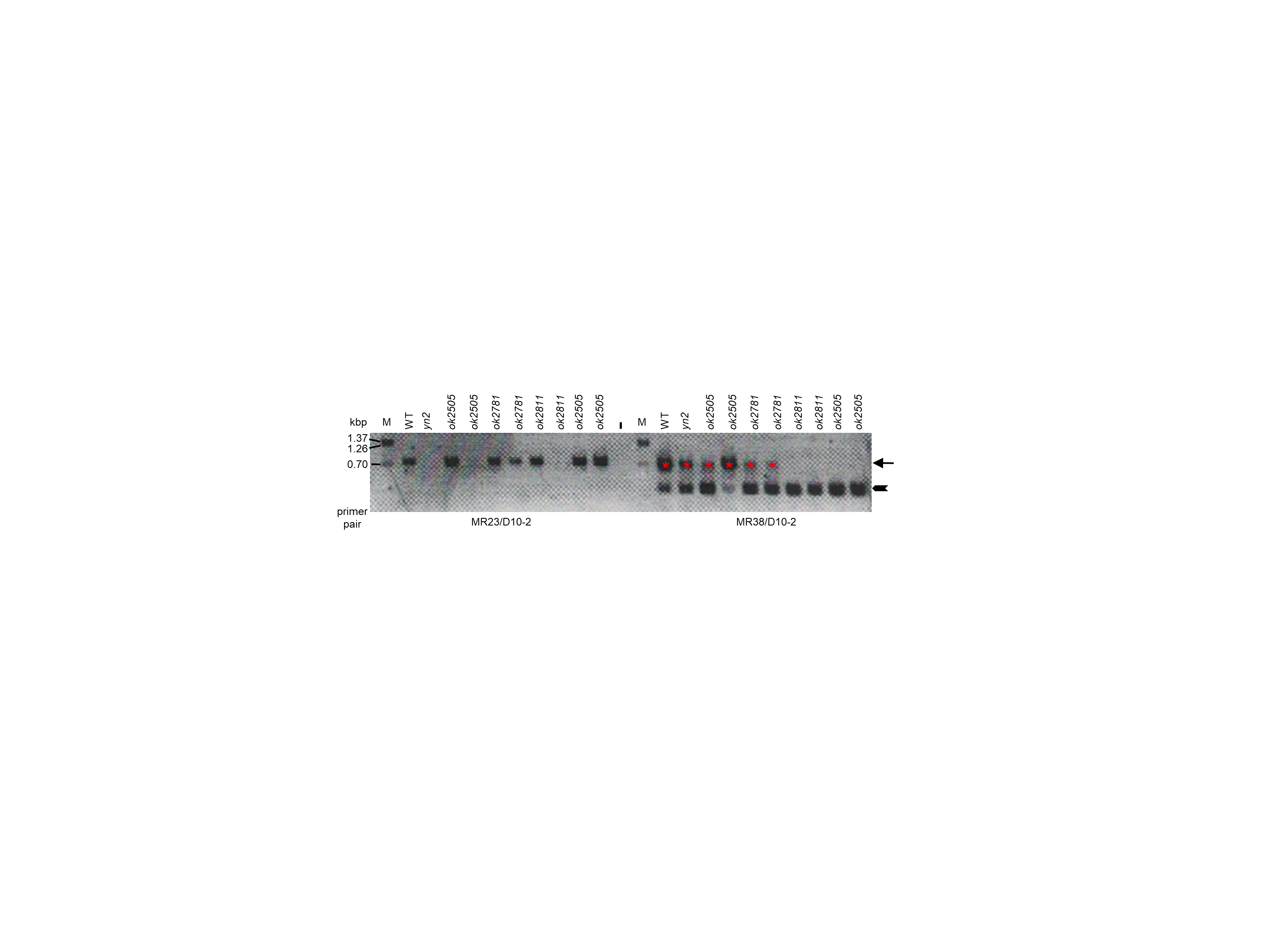

Supplement: S1 Fig — RNA from wild type (WT), flp-1 single, and daf-10 flp-1(yn2) double mutants was isolated and reverse transcribed with an oligo-dT primer. The cDNA was amplified with two primer pairs, one set (MR23/D10-2) in which one primer is contained within the yn2 deletion, but not within the flp-1 single deletion mutations (756 bp, arrow), and one set (MR38/D10-2) in which the primers are not contained within the yn2 deletion or any flp-1 single deletions (283 bp, chevron). The larger products (722 bp) in the MR38/D10-2 amplification (red asterisks) were due to DNA contamination. M = molecular weight markers. (TIF) [file pone.0189320.s002.tif]
